# Development of an Air Pollution Risk Perception Questionnaire for Running Race Runners Based on the Health Belief Model

**DOI:** 10.3390/ijerph191811419

**Published:** 2022-09-10

**Authors:** Hsueh-Wen Chow, Kuan-Lin Chen

**Affiliations:** Institute of Physical Education, Health & Leisure Studies, National Cheng Kung University, Tainan City 701, Taiwan

**Keywords:** risk perception, perceived susceptibility, perceived severity, perceived benefits, perceived barriers, cues to actions, self-efficacy, awareness of air quality

## Abstract

An increasing number of individuals participate in running races worldwide; however, running in the presence of air pollution poses health risks to runners. Therefore, developing a valid and reliable instrument is imperative to assess runners’ beliefs and perceptions regarding risks and health behaviors. This study developed a comprehensive questionnaire based on the health behavior model and relevant literature. The questionnaire was tested with 310 responses from individuals with running race experiences in Taiwan. Tests of the measurement model were conducted using reliability and confirmatory factor analysis. The results reveal that the questionnaire consists of eight constructs: perceived susceptibility, perceived severity, perceived benefits, perceived barriers, perceived self-efficacy, cues to action, health behavior intention, and awareness of air quality. The 31 items jointly accounted for 72.71% of the observed variance. All eight factors have good internal consistency, convergent, and discriminant validity with acceptable model fit indexes. Additionally, a valid translated English version of the questionnaire is provided for future research, sports agencies, or governments to explore factors that affect, or interact with, risk while running under air pollution conditions to develop management strategies.

## 1. Introduction

Physical inactivity has been considered one of the leading causes of noncommunicable diseases and has been linked with many chronic diseases and cancer [1,2]. Several initiatives and campaigns have called for programs and events to promote an active lifestyle. Road running is one such event. The growing popularity of running events worldwide has attracted a significant number of participants. Running race participants have risen from 5 million to 9.1 million in the past decade leading up to the COVID-19 pandemic [3].

In addition, the risks associated with running have also increased. There is a widespread belief that air pollution, which results from a mix of gases or particles emitted from primary sources and forming in the atmosphere, is an invisible killer that has adverse effects on health. It has been estimated that 99% of the world’s population is exposed to unhealthy levels of air pollution that exceed WHO guidelines. Air pollution is, thus, one of the greatest environmental threats to human health [4]. 

Running in air pollution presents particular hazards because of a number of factors. First, humans tend to switch from nasal to oral breathing, which weakens the nose’s ability to filter out pollutants. Second, during exercise, the body inhales more air than when sedentary, thus increasing the possibility of inhaling extra pollutants. Finally, when exercising vigorously, the body increases its ventilation rate, which means that people breathe more deeply and frequently, leading to increased deposits of pollutants in the lungs [5,6,7,8]. These factors might lead to severe cardiovascular incidents such as myocardial infarction, coronary revascularization, stroke, death from either coronary heart disease or cerebrovascular disease [9], or lung functions or cancers [10]. There is a serious negative impact. Accordingly, several studies have pointed out the necessity of further investigating the effect of air pollution on sports-related life-threatening events, particularly amongst professional and high-level amateur athletes [11].

However, air pollution is often considered to be an invisible killer, proven to have adverse health effects. To date, few studies have investigated the relationship between air pollution and running events, especially from various stakeholders such as the runners themselves, sport organizing bodies, and host cities. However, no risk management strategies are in place to prevent adverse health effects attributed to air pollution. This kind of oversight is dangerous, as many studies have identified that the risk of air pollution could have adverse health effects for individuals, such as airway inflammation, lung function alteration, elevated blood pressure, myocardial infarction, stroke, or all-cause mortality [9,12,13,14,15,16]. These effects not only occur as a result of long-term training (Miller et al., 2007) but could also take place during short-term (or one-time) racing [17].

Therefore, the overall aim of this study is to investigate the risk perceptions of air pollution and running events from three main perspectives: running event participants, host agencies or sports organizations, and governmental authorities/other stakeholders. This would help develop comprehensive risk management strategies. Considering the importance of achieving this goal, the first step is to obtain information from runners regarding their perceptions, risk awareness, and the potential health behaviors they might adopt during running races. Accordingly, this paper aims to develop a reliable and validated questionnaire to assess runners’ beliefs and perceptions of risk and health behaviors based on a grounded health behavior model. 

## 2. Materials and Methods

### 2.1. Theoretical Framework

The health belief model (HBM) is a comprehensive model commonly used to explain and predict preventative health behavior or change and maintain health behavioral patterns. It consists of seven main constructs; (1) perceived susceptibility (PSU): an individual’s perception of vulnerability to a condition and the degree to which the individual believes he or she is likely to acquire it; (2) perceived severity (PSE): the belief that the affected person might suffer serious social and medical consequences as a result of the condition; (3) perceived benefits (PBE): the belief that intervention will have a positive outcome; (4) perceived barriers (PBA): an individual believes he or she must overcome certain barriers before he or she can conduct some kind of intervention effectively; (5) perceived self-efficacy (SE): an individual evaluates how easy or difficult it is to perform a specific action or how much control he or she has over that action; and (6) cues to action (CTA): the triggers that cause an individual to act, which may be internal (symptoms of a health problem), or external (media communications, interpersonal communications, or information from healthcare providers) [18,19]. The outcome or predictive constructs in HBM are health behavior intentions (HBI) or actions that are being investigated by the research, for example, smoking behavior [20], cancer prevention [21], medication adherence [22], and exercise participation [23]. 

### 2.2. Literature Related to Running and Air Pollution Risk Perception

In addition to these seven key constructs, other variables, such as sociodemographic variables or structural variables, may also influence behavior outcomes [19]. Education, for example, has been shown to have an indirect effect on health behavior by influencing an individual’s perception of susceptibility, severity, benefits, and barriers. Individual characteristics and experiences may influence individuals’ perceptions and health behaviors in reaction to air pollution. In previous studies, variables such as gender, age, education, marital status, or respiratory symptoms have been associated with risk perceptions of air pollution. They found, for instance, that it is possible that young adults may be more aware of the health risks associated with air pollution and are, therefore, more likely to modify their exercise behaviors according to their environment [24]. Another study, however, indicates that older and more educated individuals are more aware of air pollution [25]. Researchers have found significant differences in subjective air quality assessments and perceptions of air pollution-related risks between urban and rural residents [20,21]. Additionally, studies indicate that individuals with respiratory disease or cardiovascular disease are more likely to perceive air pollution as a risk and modify their outdoor activities as a result of poor air quality [26]. 

Several studies have identified distinct differences in the motivations and behaviors of individuals when they pursue casual leisure or serious leisure [27]. While some participants in running races do so for recreational purposes, others practice running seriously and set goals for running races. Runners’ different levels of involvement might also affect their risk perceptions and behaviors, as adventure programs have demonstrated that people may be motivated just by the pleasure of doing something with little regard for the potential risks involved [28]. 

### 2.3. Questionnaire Development

To assess runners’ risk perception toward air pollution during the race, based on the HBM model described and literature review discussed above, along with an earlier focus group study with runners, several statements were generated first and then viewed by the research team as well as by several runners to confirm the clarity and logic of the questionnaire. The questionnaire consisted of (a) demographic and socioeconomic information about the participants, including age, education, residence, and income; (b) medical histories, e.g., Is there any heart/lung disease you/your family member has been diagnosed with by a physician? (c) participants’ involvement in running and participating in events related to running, such as the number of running races participated in, distances run during running races, and average monthly running training distances; (d) participants’ awareness of air quality (AAQ), inquiring if he/she experiences symptoms such as red eyes, sneeze, cough, dry throat, etc. while running under air pollution conditions; and (e) participants’ response to statements on perception in the context of running events during air pollution using the seven HBM constructs (PSU, PSE, PBE, PBA, CTA, SE, HBI). All other questions, excluding demographic, medical history, and running involvement variables, as well as five open-ended follow-up questions, were formatted on a five-point Likert scale ranging from 1 = strongly disagree to 5 = strongly agree. Several reverse questions were developed to be incorporated into the questionnaire/construct to examine respondents’ consistency in answering the questions. These questions were reverse coded for further statistical analysis.

Several experts and scholars in health behavior, public health, and education were invited to review and validate the questionnaire. They are all familiar with the Health Belief Model and air pollution issues. They were asked to evaluate the items of the questionnaire in terms of simplicity, clarity, relevance, and necessity in reflecting each construct in HBM and other variables related to the purpose of this study. The necessary changes and corrections were applied to the questionnaire’s text based on their opinions to determine the content validity. The overall questionnaire items and responses categories are presented in Appendix A.

### 2.4. Data Collection and Ethical Approval

To administer the questionnaire efficiently without geographic limitations, the questionnaire was transformed into an online format using the Google Forms application. Online questionnaires were distributed through running-related social media groups, such as Facebook and Line, and distributed to potential runners through personal networks. Participants were allowed to win NTD200 (about $7 dollars) as an incentive. Data were collected between April and June 2021 in Taiwan. 

On the first page of the questionnaire, the researcher described the purpose of the study and contact information for the research team. Participants were assured of anonymity and confidentiality for their responses. By clicking on the ‘agree’ button with their consent to participate in this study, the respondents were directed to the main section of the questionnaire. All materials and study procedures were approved by National Chen Kung University Research Ethics Committee (REC) (108–507), where the study was conducted.

As the first filter question, respondents were asked if they had previously participated in running races. If the participant replied that they had never participated in any road running race before, the page was automatically redirected to a message of appreciation for participation. For respondents who completed the entire questionnaire, the average time was approximately 20–25 min. The minimum sample size was 385, assuming a 95% confidence level, a margin of error (confidence interval) of ±5%, and a 0.5 standard deviation. According to Kline [29], a good rule of thumb is to have at least 200 samples or 5–10 times as many subjects as the number of variables. 

### 2.5. Data Analysis

The responses from the online questionnaire from the Google form were first exported into a csv file. Next, responses with no prior experience with running events and inconsistent responses were deleted. The data were then imported into SPSS and Amos (IBM SPSS Statistics for Windows, Version 27.0. IBM Corp, Armonk, NY, USA) for further data analysis. 

To begin with, reliability analysis examines the intercorrelations between the items within each construct, i.e., the extent to which the items measure the same underlying concept. To assess the instrument’s reliability, 100 participants participated in a pilot study. The internal consistency of each construct was determined using Cronbach’s alpha. 

The questionnaire was developed based on a widely used HBM model, so we did not perform exploratory factor analysis. Instead, the confirmatory factor analysis (CFA) was used to verify the suitability of each item to measure the intended construct, both individually and collectively, for the final sample. To describe the characteristics of the sample and the variables in the study, a descriptive analysis was performed. A composite score was calculated for each construct based on the means of the responses to the items that comprised each construct. Therefore, we were able to compare scores across constructs. High scores indicated stronger feelings regarding that particular construct. As a result, higher scores indicate greater perceived susceptibility, severity, etc. In addition, Pearson’s correlation coefficient was conducted to examine the intercorrelation among constructs.

Structure equation modeling (SEM) was used to estimate and test the proposed measurement model. As suggested by Kline [29], multiple fit indices should be assessed in combination to assess goodness of fit to provide a holistic view in terms of sample size, model complexity and other relevant issues. The model’s fit was examined by using the maximum likelihood chi-square values/degrees of freedom ratio (CMIN/DF), the goodness of fit index (GFI), the comparative fit index (CFI), the Tucker–Lewis index (TLI), and the root mean square error of approximation (RMSEA). The conventional thresholds for model fit are: CMIN/DF < 3 good (<5 acceptable), GFI value >  0.9 good (>0.8 sometimes permissible), CFI value > 0.9, TLI value > 0.9, and RMSEA value <  0.05 good (0.05~0.10 is moderate) [30].

### 2.6. Questionnaire Translation

The questionnaire was first developed in Chines as it was administered in Taiwan. In order for this questionnaire to be used worldwide or for future cross-cultural research, the forward-backward-forward translation method was used to translate the questionnaire from Chinese to English and then from English to Chinese again. Two translators fluent in both English and Chinese undertook the translation process [31]. They were all experienced researchers. We also distributed the translated questionnaire to our research team and three external experts from other countries to get feedback on each question’s difficulty, clarity, and appropriateness. Apart from some demographic variables whose answers should be adjusted based on the country’s standard or cultural sensitivity, all other questions are clearly defined. 

## 3. Results

### 3.1. Descriptive Statistics of the Sample and Investigated Constructs

A total of 448 online responses were received; after removing those who did not participate in a running race and those whose responses were inconsistent; 310 responses were retained for further analysis. Among these respondents, 189 are male (59%) and 127 are female (41%). Most of the respondents are in the age group of 31–40 years old (33.9%). Almost half (44.8%) of them have educational levels below university/college, while the other half hold a university or college degree or above. The respondents’ income is fairly distributed among the four response categories. Most of them reside in southern Taiwan (51.6%). In terms of their running experience, 78.4% have only participated in races less than five times. Most of the respondents reported that they participated in races of less than 5 km (70.3%) and that their monthly running practice was less than 25 km (76.8%). Among the respondents, 17 (5.5%) reported having a heart condition and four (1.3%) reported a lung condition. A total of 47 (15.2%) and 19 (6.1%) respondents report that a family member has been diagnosed with heart or lung disease, respectively (Table 1). 

The responses to all statements in the HBM constructs and AAQ are presented as mean and standard deviation for each question and a composite score for each construct (Table 2). In this study, respondents indicated the highest level of agreement on the PSE construct (mean: 4.307), in which they felt that participating in a running race during high levels of air pollution will result in significant negative health consequences. As the second-highest agreed to construct, CTA (mean: 4.26) indicates respondents agree that stimulation through family, friends, or media channels regarding air pollution information may serve as a cue to take appropriate action. The lowest agreement construct is SE (mean: 2.960). Runners believed that they had little control over the action that they would like to take. 

### 3.2. Reliability and Validity of the Study Constructs

Overall, 71 questions were developed and both the Chinese and English questions can be found in Appendix A. Fifty-one items were generated using a 5-point Likert scale measuring all constructs. Then, we examined the measurement model based on indicator reliability from the 100 pilot test samples. Five items were removed due to their low item reliability contributing to their respective construct. The pilot test results in 46 items loaded on seven HBM constructs and an AAQ construct with the following acceptable Cronbach alpha values: PSU = 0.73, PSE = 0.90, PBE = 0.70, PBA = 0.64, CTA = 0.77, SE = 0.71, HBI = 0.76, AAQ = 0.90. At the next stage, 310 samples were tested using the CFA measurement model. Factor loadings are used to determine the strength of the relationship between the item and the construct it falls within. According to Hair et al. [32], a factor loading less than 0.55 is deemed unsuitable. As such, 15 items were removed. In addition, the standard criteria of construct composite reliability (CR) > 0.6 and the average variance extracted (AVE) > 0.036 were also examined [33] and are presented in Table 2. The results ensure that the overall measurement model demonstrates adequate discriminant validity; each construct is distinctive from the others. We further examine the construct multicollinearity by correlation matrix, tolerance and variance inflation factor (VIF) values based on the acceptable rule of thumb criteria (Pearson < 0.8, tolerance close to 1, VIF < 2.5) (see Table 3 for Pearson correlation matrix, tolerance and VIF). The final items for each construct are perceived susceptibility (PSU: 3 items), perceived severity (PSE: 4 items), perceived benefits (PBE: 3 items), perceived barriers (PBA: 3 items), cues to action (CTA: 5 items), self-efficacy (SE: 3 items), health behavior intentions (HBI: 2 items), and awareness of air quality (AAQ: 8 items), all of which result in a total of 31 items. The variance explained by these constructs is 72.71%.

### 3.3. Measurement Model of HBM

**Figure 1:**
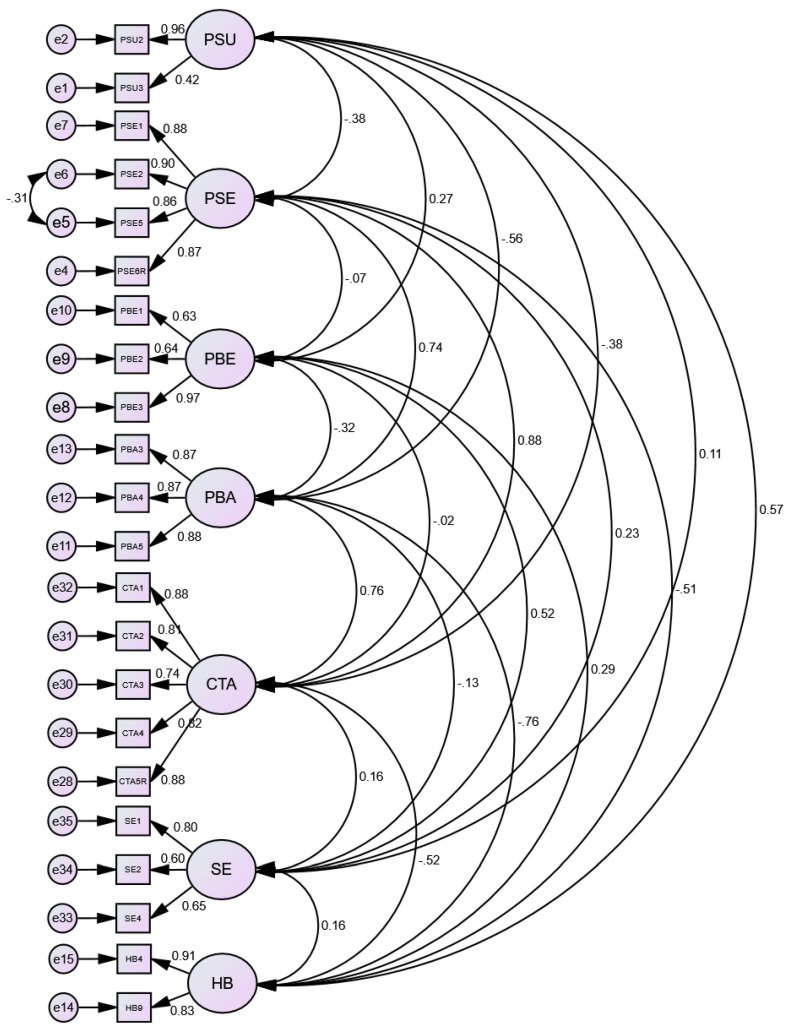
CFA of HBM measurement model.

CFA from AMOS software was tested on the 23 final items of the seven constructs in the HBM model to determine its model fit for measurement. Some model fit indices were initially not good. We attempted to improve the model by indicate covariances between errors (e5 & e6) and remove a problematic variable (PSU 1). By doing so, the model fit indeed improved a little bit. The model indicated an acceptable good fit to the data in the following indices (CMIN/DF = 2.47; GFI = 0.864, RMSEA = 0.069). but not on CFI = 0.599 and TLI = 0.505. The standardized parameter estimates were presented in Figure 1. 

## 4. Discussion

This study aims to develop a comprehensive questionnaire to assess factors influencing runners’ health beliefs, risk perception, and adaptive behaviors while participating in air polluted running races. The study was developed from the HBM model and includes important factors and characteristics affecting the runners’ health beliefs and risk perceptions while facing air pollution during races. 

Several items were removed during the process of reliability and validity examination. For example, for the outcome HBI construct, five items were omitted from the original seven questions, which resulted in an inability to generate a reliable construct. This might be attributable to the fact that there are various degrees of intentions when runners decide to face polluted air races. The initial items include using proactive measures, such as wearing masks or taking some nutrients, which might not be the same as withdrawing from the race. Further research could treat each outcome variable as an individual dependent variable instead of a single construct. This will allow a clear picture of the different runners’ characteristics and health belief factors in predicting their various health behavior intentions. Additionally, only two items remained in the PSU construct. In the original questionnaire, four questions were asked about the likelihood that runners would encounter air pollution during a running race (e.g., PSU1: I often encountered air pollution when I participated in a road running race in the past.). No health-related questions were asked of the subjects in the four questions. It is recommended that this construct should, in the future, incorporate items related to individuals’ perceptions of polluted air’s impact on their health. For example, my health will likely get worse because of participating in an air-polluted race event.

The Pearson correlation among constructs and the CFA model reveals that several constructs are inter-correlate. The strongest positive relationships are between perceived severity (PSE) to cues to action (CTA) (r = 0.586, *p* < 0.01) and perceived barriers to cues to action (r = 0.508, *p* < 0.01). This indicates that the triggers or cues that prompt runners’ to take a health behavior intention are highly related to their belief that the serious negative impact of air pollution on races is significant and imposes perceived barriers. It might also imply that, contrary to the traditional HBM model, CTA directly influences health behavior. Because media information is so fast and easy, CTA may be an important antecedent or determinant of a runner’s perceived susceptibility or any perceived barriers. A significant positive relationship also exists among PSExPSU, PBAxPSE, PBAxPBE, PBExSE, CTAxSE, PSExHBI, PBExHBI, CTAxHBI, SExHBI, PSUxAAQ, PSExAAQ, PBExAAQ, HBxAAQ. The significant negative relationship lies between PSUXSE and PBAXAAQ. 

Although the questionnaire was developed from a theoretical HBM model with rigorous psychometric testing, results of the CFA model produced only an acceptable model fit. This might be attributable to the fact that this study did not test the items from exploratory factor analysis (EFA) due to the established HBM model. Therefore, it is recommended that future research incorporating the HBM model should start with EFA in order to assess the factor structure within the study context. Likewise, the AAQ construct is an additional construct that was developed based on the study context of this study. Whether this construct should be incorporated into the original HBM model to form a second order measurement model or treated as an independent antecedent, moderate, or mediate construct in predicting health behaviors. This study established the validity and reliability of the instruments using Taiwanese data, but future research should examine how this tool might be applied across cultures to conduct cross culture comparisons. 

Other health behavior models or theories might also be considered in future research, such as the theory of planned behavior (TPB) [34] or the self-determination theory (SDT) [35]. According to TPB, runners’ behavior may be heavily influenced by their beliefs (whether participating in an air pollution race is harmful to their health), subjective normative (how important referents agree/disagree with participating in air pollution races), and control beliefs (runners’ perceptions of their ability to control air pollution’s impact on their race participation). The SDT emphasizes the psychological needs and motivations of human behavior, particularly autonomy. Given that many runners have demonstrated the characteristics of being motivational, serious, autonomous, and resilient to their pursuit of this activity [36,37]. SDT could be applied to determine whether running race participants are motivated intrinsically or extrinsically. When confronted with air pollution, their behavior is also influenced by three fundamental psychological needs: autonomy, competence, and relatedness. Examining which model could best explain runners’ behavior might be helpful.

## 5. Conclusions

This paper aimed to develop a questionnaire to assess runners’ risk perception and adaptive behaviors when participating in race events associated with air pollution. The questionnaire was framed from the HBM model and related literature with a holistic assessment of an individual’s personal demographic and medical histories, running involvement, and the level of his/her awareness of air pollution symptoms and health behaviors. Results indicate that questionnaires are acceptable tools with acceptable validity and reliability for some measures. Also, we provided a detailed discussion on how to modify the questionnaire in the future or how to use other health behavior models. It is nevertheless clear from our results that this instrument might provide useful insights. The findings from our single empirical setting in Taiwan provide the foundation for this research agenda. In the future, work in different settings could enable us to develop an instrument that is more generalizable and can be used in a variety of settings. 

This questionnaire is valuable for future research, while sports agencies and governments may use it to explore the factors that affect or interact with risk while running under air pollution conditions. For example, sports agencies can use these tools to better understand risk management boundaries and develop health risk management strategies tailored for runners with different backgrounds. Future researchers will assist local governments in identifying high-risk populations, improving health awareness, and getting runners to take protective measures to reduce pollution effects.

## Figures and Tables

**Table 1:** Sociodemographic variables and medical history from the participants.

Variable	Category	No.	Percentage
Gender	(1) Male	183	59
	(2) Female	127	41
Age	(1) Under 30	66	21.3
	(2) 31 to 40	105	33.9
	(3) 41 to 50	72	23.2
	(4) Over 51	67	21.6
Educational level	(1) Under senior high school	139	44.8
	(2) University/College	143	46.1
	(3) Graduate school	28	9
District of residence	(1) North Taiwan	33	10.6
	(2) Middle Taiwan	116	37.4
	(3) South Taiwan	160	51.6
	(4) East Taiwan	1	0.3
Personal annual income	(1) Under NTD 300 K	63	20.3
	(2) NTD 310 K to 600 K	95	30.6
	(3) NTD 610 K to 900 K	80	25.8
	(4) Over NTD 910 K	72	23.2
Number of road race participated	(1) Under 5 times	243	78.4
	(2) 6 to 10 times	39	12.6
	(3) Over 11 times	28	9
Competition distance of road race	(1) Under 5 km	218	70.3
	(2) 6 to 10 km	39	12.6
	(3) Over half marathon	51	16.5
	(4) Other	2	0.6
Amount of practice for each time	(1) Less than 1 km	156	50.3
	(2) 1 to 10 km	121	39
	(3) Over 11 km	33	10.6
Average running distance in the last mouth	(1) Under 25 km	238	76.8
	(2) 26 to 50 km	35	11.3
	(3) Over 51 km	37	11.9
Having heart disease	(1) Yes	17	5.5
	(2) No	293	94.5
Family history of heart disease	(1) Yes	47	15.2
	(2) No	263	84.8
Having lung disease	(1) Yes	4	1.3
	(2) No	306	98.7
Family history of lung disease	(1) Yes	19	6.1
	(2) No	291	93.9

**Table 2:** Mean, standard deviation, Cronbach’s α values, factor loading, composite reliability (CR), the average variance extracted (AVE) of questionnaire items and constructs from the pilot test sample and the final sample.

		Pilot Test Sample (*N* = 100)			Final Sample (*N* = 310)							
Construct	Item	Cronbach’s α if Item Deleted	Decision (V: Keep; X: Removed)	Construct Reliability	Factor Loading	Decision	CR	AVE	Item Mean	Item Std.	Construct Mean	Construct Std.
PSU	PSU1	0.59	V	0.73	0.58	V	0.63	0.36	3.05	0.66	3.13	0.52
	PSU2	0.71	V		0.6	V			2.52	0.91		
	PSU3	0.70	V		0.63	V			3.35	0.82		
	PSU4R	0.68	V			X			3.61	0.82		
PSE	PSE1	0.88	V	0.90	0.91	V	0.93	0.77	4.6	0.79	4.31	0.56
	PSE2	0.87	V		0.88	V			4.55	0.81		
	PSE3	0.88	V			X			3.46	0.80		
	PSE4	0.90	V			X			4.07	0.59		
	PSE5	0.88	V		0.86	V			4.65	0.68		
	PSE6R	0.88	V		0.86	V			4.51	0.89		
PBE	PBE1	0.59	V	0.70	0.63	V	0.74	0.49	3.14	0.73	3.13	0.59
	PBE2	0.61	V		0.67	V			3.11	0.77		
	PBE3	0.50	V		0.79	V			3.14	0.69		
	PBE4	0.58	X			D						
	PBE5	0.65	X			D						
PBA	PBA1	0.59	V	0.64		X	0.88	0.7	3.75	0.88	3.53	0.66
	PBA2	0.56	V			X			1.73	1.08		
	PBA3	0.61	V		0.79	V			3.76	1.44		
	PBA4	0.53	V		0.93	V			4.17	1.12		
	PBA5	0.57	V		0.79	V			4.22	1.10		
	PBA6R	0.64	X			D						
CTA	CTA1	0.71	V	0.77	0.86	V	0.89	0.62	4.45	0.89	4.26	0.76
	CTA2	0.74	V		0.79	V			4.42	0.91		
	CTA3	0.72	V		0.72	V			3.66	0.83		
	CTA4	0.77	V		0.68	V			4.52	0.80		
	CTA5R	0.71	V		0.87	V			4.26	1.11		
SE	PSE1	0.17	V	0.71	0.58	V	0.7	0.45	3.07	0.71	2.96	0.57
	PSE2	0.14	V		0.76	V			2.92	0.70		
	PSE4	0.12	V		0.65	V			2.89	0.74		
	PSE6R	0.48	X			D						
	PSE7	0.48	X			D						
HBI	HBI1	0.68	V	0.76		X	N/A	N/A	3.94	0.70	3.48	0.51
	HBI2	0.77	V			X			3.78	0.86		
	HBI4	0.72	V		N/A	V			2.67	1.06		
	HBI5	0.80	V			X			3.93	1.38		
	HBI7R	0.71	V			X			3.71	0.86		
	HBI8	0.70	V			X			3.75	0.87		
	HBI9	0.69	V		N/A	V			2.6	1.02		
AAQ	AAQ1	0.89	V	0.90		X	0.92	0.61	3.95	0.61	3.40	0.70
	AAQ2	0.90	V		0.8	V			1.72	1.09		
	AAQ4	0.89	V		0.8	V			2.68	1.03		
	AAQ5	0.90	V			X			3.98	0.77		
	AAQ6	0.89	V		0.77	V			2.44	0.88		
	AAQ7	0.88	V			X			2.64	1.06		
	AAQ8	0.89	V		0.82	V			2.54	1.03		
	AAQ9	0.88	V		0.86	V			2.63	1.01		
	AAQ10	0.88	V		0.85	V			2.95	0.88		
	AAQ11	0.89	V		0.82	V			3.01	0.87		
	AAQ12	0.89	V		0.84	V			2.79	0.88		
	AAQ13	0.89	V			X			3.7	0.78		
	AAQ14	0.88	V			X			4.2	1.01		

**Table 3:** Pearson correlation, tolerance and VIF among investigated constructs.

	PSU	PSE	PBE	PBA	CTA	SE	HBI	AAQ
PSU	1	.154 **	.096	−.098	.000	−.112 *	.086	.382 **
PSE		1	.048	.237 **	.586 **	−.046	.336 **	.195 **
PBE			1	−.185 **	.028	.290 **	.305 **	.314 **
PBA				1	.508 **	.031	−0.024	−.407 **
CTA					1	.159 **	.343 **	−.023
SE						1	.288 **	−.072
HBI							1	.289 **
AAQ								1
Tolerance	0.836	0.592	0.799	0.574	0.479	0.836		0.608
VIF	1.196	1.690	1.252	1.741	2.089	1.196		1.644

Note: * *p* < 0.05, ** *p* < 0.01.

## Data Availability

Summaries of the data are available on reasonable request from the corresponding author. The data are not publicly available due to containing potentially identifiable information about participants.

## Appendix A

Runners’ risk perceptions encountering air pollution running race questionnaire (Chinese and English versions).

**Construct**	**Questions in Chinese**	**Question in English**	**Response Options**
Consent question	點擊以下同意按鈕代表您已滿18歲並了解本研究目的並同意參與此調查	By clicking “I agree” below you are indicating that you are at least 18 years old, have read and understood this consent form and agree to participate in this research study.	1. I agree to the terms and conditions/2. I disagree
Filter question	請問您是否有參與過任何路跑賽事?	Have you participated in any road running races?	1. Yes/2. No-End of participation
Sociodemographic	請問您的性別?	Your gender?	1. Male/2. Female/3. Other
	請問您民國幾年出生?	In what year were you born?	Type year born
	請問您的最高學歷?	What is your highest education level?	1. Elementary school/2. Junior high school/3. High school/4. University/College/5. Graduate school/6. Doctoral Program
	請問您的居住國家?	Which country do you live in?	Short answer (text)
	請問您的居住鄉鎮市區?	Which city/town do you live in?	Short answer (text)
	請問您的個人年收入?	What is your annual personal income (gross)?	1. Under 1000 USD/2. 1001~2000 USD/3. 2001~3000 USD/4. 3001~4000 USD/5. 4001~5000 USD/6. Over 5000 USD
Running experience	請問您參與路跑賽事大概的總次數?	How many times have you participated in road races?	Type number.
	請問您大部分參與賽事的參賽距離組別為?	When you participated in a running race, what is the distance that you ran most of the time?	1. Under 5 K/2. 5 K/3. 10 K/4. Half Marathon/5. Ultra-Half Marathon (22~41 K)/6. Marathon/7. Ultra-Marathon (Over 43 K)/8. Others
	請問您一般練習大概的月跑量(公里)?	Generally, what is your average running distance (km) in the last months?	1. less then 25 K/2. 26–50 K/3.51–100 K/4. 101–200 K/5. over 201 K
Medical history	您是否經醫師診斷患有任何心血管疾病?	Do you have any heart diseases diagnosed by a physician? (mark all that apply)	1. None/2. Coronary artery syndrome/3. Stroke/4. Hypertensive heart disease/5. Rheumatic heart disease/6. Aneurysm/7. Cardiomyopathy/ 8. Atrial fibrillation/9. Congenital heart disease/10. Endocarditis/11. Peripheral arterial obstructive disease/12. Other heart diseases/13. other___________(specify)
	您的家人是否經醫師診斷患有任何心血管疾病?	Does any of your family members have heart diseases diagnosed by a physician? (mark all that apply)	
	您是否經醫師診斷患有任何肺部疾病?	Do you have any lung diseases diagnosed by a physician? (mark all that apply)	1. None/2. Emphysema/3. Asthma/4. Tuberculosis/5. Chronic obstructive pulmonary disease/6. Lung cancer/7. Chronic bronchitis/8. Other lung disease/9.other____________(specify)
	您的家人是否經醫師診斷患有任何肺部疾病?	Does any of your family members have any lung diseases diagnosed by a physician? (mark all that apply)	
Perceived susceptibility	PSU1. 我過去在參與路跑賽事時，經常是有空氣汙染的狀況。	I often encountered air pollution when I participated in a road running race in the past.	Likert scale (1. strongly disagree to 5 strongly agree)
	PSU2. 我認為我在報名路跑賽事時，已經可以預期到空氣汙染可能會發生。	I anticipate that air pollution could be a problem when I sign up for a running race.	
	PSU3. 我認為我未來在參與路跑賽事時有很高機率會碰到空氣汙染。	I think I have a high chance of encountering air pollution when participating in road running races in the future.	
	PSU4R. 我過去在參與路跑賽事時，不常有空氣汙染的狀況。	From my experience, it was very unlikely that air pollution is high when participating in road races.	
Perceived severity	PSE1. 我認為在空氣汙染的狀況下參與路跑賽事會影響到我的健康。	I think participating in road running races under air pollution conditions will affect my health.	Likert scale (1. strongly disagree to 5 strongly agree)
	PSE2. 我認為在空氣汙染的狀況下參與路跑賽事會影響到我的運動表現。	I think participating in road running races under air pollution conditions will influence my athletic performance negatively.	
	PSE3. 我認為在空氣汙染的狀況下參與路跑賽事會讓我的心情較為負面。	I think participating in road running races under air pollution conditions will distress my mood.	
	PSE4. 我認為在空氣汙染的狀況下參與路跑賽事會影響到我的賽事體驗與感受。	I think participating in road running races under air pollution conditions will disturb my race experience.	
	PSE5. 我認為長期在空氣汙染的狀況下參與路跑賽事會導致心肺功能受到負面影響。	I think that the long-term engagement in road running races under air pollution could lead to adverse cardiovascular functions.	
	PSE6R. 我認為在空氣汙染的狀況下參與路跑賽事對我不會有嚴重的影響。	I don’t think participating in road races in air pollution conditions will seriously impact my health.	
Perceived benefits	PBE1. 因空氣汙染而避免參與路跑賽事，讓我有更多時間能夠陪伴我的朋友或家人。	If I withdraw from running races because of the air pollution, I could have more time to accompany my friends or family.	Likert scale (1. strongly disagree to 5 strongly agree)
	PBE2. 因空氣汙染而避免參與路跑賽事，讓我能夠有機會在當地從事其他活動或旅遊。	If I withdraw from running races because of the air pollution, I could have more time to engage in other activities or travel locally.	
	PBE3. 因空氣汙染而避免參與路跑賽事，讓我能夠彈性調整原本規劃好的預定行程。	If I withdraw from running races because of the air pollution, I could have the flexibility to adjust my schedule.	
	PBE4. 因空氣汙染而避免參與路跑賽事，讓我能夠避免掉空氣汙染所帶來的身體不適。	If I withdraw from running races because of the air pollution, I could avoid the physical discomfort caused by air pollution.	
	PBE5. 因空氣汙染而避免參與路跑賽事，長久而言對我的健康是有益的。	If I withdraw from running races because of the air pollution, it would be beneficial to my health in the long run.	
Perceived barriers	PBA1. 我認為因為空氣汙染而避賽，會讓我損失我的報名費。	If I withdraw from running races because of air pollution, I will lose my registration fee.	Likert scale (1. strongly disagree to 5 strongly agree)
	PBA2. 我認為因為空氣汙染而避賽，會讓我先前的練習通通都白費。	If I withdraw from running races because of the air pollution, my previous efforts for practicing running will be in vain (useless).	
	PBA3. 我認為因為空氣汙染而避賽，有違運動家精神積極展現的態度。	I think it is against (contrary to) sportsmanship if I withdraw from running races because of the air pollution.	
	PBA4. 我認為因為空氣汙染而退賽，會打亂我既有的預定行程。	My original plan will be disrupted if I withdraw from running races because of the air pollution.	
	PBA5. 我認為因為空氣汙染而避賽，會讓我的心情與興致受到負面影響。	I feel bad (terrible) if I withdraw from running races because of the air pollution.	
	PBA6R. 我不會因為空氣汙染的影響而掙扎是否參賽 。	I won’t struggle taking part in running race because of air pollution.	
Cues to action	CTA1. 我會透過網路媒體或其他管道(如APP) 取得空氣品質的相關訊息。	I obtain information about air quality through websites, apps or other media.	Likert scale (1. strongly disagree to 5 strongly agree)
	CTA2. 我的家人或朋友會提醒我注意戶外空氣品質的狀況。	My family or friends remind me of the outdoor air quality.	
	CTA3. 我的網路社群或通訊軟體社團不定時會談論到關於戶外空氣品質的狀況。	My social media community comments about the outdoor air quality.	
	CTA4. 我有耳聞到長期在空氣汙染環境下可能會導致身體危害的案例。	I have heard of someone getting ill after long-term exposure to air pollution	
	CTA5R. 我平時不會特別注意與空氣品質有關的訊息。	I rarely pay attention to air quality.	
Perceived self-efficacy	SE1. 我能夠有效地避免在空氣汙染的狀況下參與路跑賽事。	I can avoid participating in road races under air pollution.	Likert scale (1. strongly disagree to 5 strongly agree)
	SE2. 我在路跑賽事參賽前能夠有效地準備充足的預防措施來避免空氣汙染可能帶來的傷害。	I can prepare protective measures to avoid possible harm caused by air pollution before participating in the road race.	
	SE3. 承上題，若您有採取預防措施，請簡述您的預防措施，謝謝(無則免填)。	Follow-up, if any protective measures have been taken, please describe them briefly, thank you. (No need to fill if none)	Open-ended question
	SE4. 我在參與路跑賽事當下若空氣汙染過度影響賽事，我能夠有效地藉由預防措施來保護自己。	When I am participating in road races, if air pollution affects the races excessively, I can effectively protect myself by taking preventive measures.	
	SE5. 承上題，若您有採取預防措施，請簡述您的預防措施，謝謝(無則免填)。	Follow-up, if any protective measures have been taken, please describe them briefly, thank you. (No need to fill if none)	Open-ended question
	SE6R. 在空氣汙染的狀況下參與路跑賽事，我會難以抉擇是否參賽。	Participating in road races under the condition of air pollution will make it difficult for me to decide whether to participate in the race	
	SE7. 當我參加路跑賽事時，如果空氣污染對比賽的影響太大，我會立即棄賽。	If air pollution significantly impacts my performance during a road race, then I’ll give up immediately.	
Health Behavior Intention	HBI1. 當我在參賽前得知參賽當下會有空氣汙染的警告(AQI紅色警告)，我將會避免參賽。	I will avoid the road race when I learn about air pollution warnings (air quality index (AQI)) before the road race.	Likert scale (1. strongly disagree to 5 strongly agree)
	HBI2. 當我在參賽當下受到空氣汙染影響，我會採取預防措施(如:戴口罩、使用毛巾遮掩口鼻等)。	When I realize that I am affected by air pollution during the race, I will take preventive measures such as wearing masks or using towers to cover nose/mouth)	
	HBI3. 承上題，若您有採取預防措施，請簡述您的預防措施，謝謝(無則免填)。	Follow-up, if any protective measures have been taken, please describe them briefly, thank you. (No need to fill if none)	Open-ended question
	HBI4. 當我在參賽當下受到空氣汙染影響，我會快速離開現場。	When I realize that I am affected by air pollution during the race, I will leave immediately.	
	HBI5. 當我在參賽當下受到空氣汙染影響，賽後我會補充保養肺部之營養品。	When I am affected by air pollution during the road race, I will take some nutrients after the road race to keep my lung health.	
	HBI6. 承上題，若同意請簡述您補充之營養品名，謝謝 (若無則免填)	Follow-up, if any nutrients have been taken, please describe them briefly, thank you. (No need to fill if none)	Open-ended question
	HBI7R. 當我在參賽當下受到空氣汙染影響，我也完全不會迴避。	When I am affected by air pollution during the road race, I will still participate.	
	HBI8. 當我在比賽前一天收到比賽當天會有空氣汙染的警告(AQI紅色警告)，我會取消參賽。	When I learn that there will be an air pollution warning on the day of the race before the event, I will cancel my registration.	
	HBI9. 當我在比賽當天收到空氣汙染超過最低標準的警告(AQI紅色警告)，我會立即退賽。	When a warning of air pollution exceeds the minimum standard on the day of the event (>AQI red color), I will withdraw my participation.	
Awareness of air quality風險感知	AAR1. 如果空氣品質不好，我會注意到。	I will notice the air quality if it is bad.	Likert scale (1. strongly disagree to 5 strongly agree)
	AAR2. 我會對空氣品質敏感的主要原因是因為我個人的病史(如:氣喘)。	I am sensitive to air pollution because I have a personal medical history.	
	AAR3. 承上題，若同意請簡述您個人的病史(若無則免填)。	Follow-up, if you are willing, please give a brief summary of your medical history. (No need to fill if none)	Open-ended question
	AAR4. 在空氣汙染的狀況下在戶外跑步，我會聞到難聞的氣味。	While running under air pollution conditions, I will smell an unpleasant smell outdoors.	
	AAR5. 在空氣汙染的狀況下跑步，我注意到天空總是灰濛濛的。	While running under air pollution conditions, I will notice that the sky is cloudy.	
	AAR6. 在空氣汙染的狀況下跑步，我的眼睛會紅腫癢。	While running under air pollution conditions, I will have “red” eyes.	
	AAR7. 在空氣汙染的狀況下跑步，我的鼻子會受到刺激。	While running under air pollution conditions, I will suffer from nose irritation.	
	AAR8. 在空氣汙染的狀況下跑步，我會打噴嚏。	While running under air pollution conditions, I will sneeze.	
	AAR9. 在空氣汙染的狀況下跑步，我的喉嚨會異常乾燥。	While running under air pollution conditions, I will have a dry throat.	
	AAR10. 在空氣汙染的狀況下跑步，我會咳嗽。	While running under air pollution conditions, I will cough.	
	AAR11. 在空氣汙染的狀況下跑步，我會容易呼吸困難。	While running under air pollution conditions, I will have difficulty breathing.	
	AAR12. 在空氣汙染的狀況下跑步，我會頭疼。	While running under air pollution conditions, I will get a headache.	
	AAR13. 在空氣汙染的狀況下跑步，我喝水的量會比平常來的更多。	While running under air pollution condition, I will drink more water than usual.	
	AAR14. 在空氣汙染的狀況下跑步，我更容易疲勞且耐力減少。	While running under air pollution condition, I will get fatigued easily.	
